# From affinity selection to kinetic selection in Germinal Centre modelling

**DOI:** 10.1371/journal.pcbi.1010168

**Published:** 2022-06-03

**Authors:** Danial Lashgari, Elena Merino Tejero, Michael Meyer-Hermann, Mathieu A. F. Claireaux, Marit J. van Gils, Huub C. J. Hoefsloot, Antoine H. C. van Kampen

**Affiliations:** 1 Bioinformatics Laboratory, Epidemiology and Data Science, Amsterdam Public Health research institute, Amsterdam Institute for Infection and Immunity, Amsterdam, the Netherlands; 2 Department for Systems Immunology and Braunschweig Integrated Centre of Systems Biology, Helmholtz Centre for Infection Research, Braunschweig, Germany; 3 Institute for Biochemistry, Biotechnology and Bioinformatics, Technische Universität Braunschweig, Braunschweig, Germany; 4 Department of Medical Microbiology and Infection Prevention, Amsterdam UMC, University of Amsterdam, Amsterdam Institute for Infection and Immunity, Amsterdam, the Netherlands; 5 Biosystems Data Analysis, Swammerdam Institute for Life Sciences, University of Amsterdam, Amsterdam, the Netherlands; Imperial College London, UNITED KINGDOM

## Abstract

Affinity maturation is an evolutionary process by which the affinity of antibodies (Abs) against specific antigens (Ags) increases through rounds of B-cell proliferation, somatic hypermutation, and positive selection in germinal centres (GC). The positive selection of B cells depends on affinity, but the underlying mechanisms of affinity discrimination and affinity-based selection are not well understood. It has been suggested that selection in GC depends on both rapid binding of B-cell receptors (BcRs) to Ags which is kinetically favourable and tight binding of BcRs to Ags, which is thermodynamically favourable; however, it has not been shown whether a selection bias for kinetic properties is present in the GC. To investigate the GC selection bias towards rapid and tight binding, we developed an agent-based model of GC and compared the evolution of founder B cells with initially identical low affinities but with different association/dissociation rates for Ag presented by follicular dendritic cells in three Ag collection mechanisms. We compared an Ag collection mechanism based on association/dissociation rates of B-cell interaction with presented Ag, which includes a probabilistic rupture of bonds between the B-cell and Ag (Scenario-1) with a reference scenario based on an affinity-based Ag collection mechanism (Scenario-0). Simulations showed that the mechanism of Ag collection affects the GC dynamics and the GC outputs concerning fast/slow (un)binding of B cells to FDC-presented Ags. In particular, clones with lower dissociation rates outcompete clones with higher association rates in Scenario-1, while remaining B cells from clones with higher association rates reach higher affinities. Accordingly, plasma cell and memory B cell populations were biased towards B-cell clones with lower dissociation rates. Without such probabilistic ruptures during the Ag extraction process (Scenario-2), the selective advantage for clones with very low dissociation rates diminished, and the affinity maturation level of all clones decreased to the reference level.

## Author summary

Adaptive immunity is one of the vital defence mechanisms of the human body to fight virtually unlimited types of pathogens by producing antigen-specific high-affinity antibodies that bind to pathogens and neutralise them or mark them for further elimination. Affinity is a quantity used to measure and report the strength of interaction between antibodies and antigens that depends both on how fast antibodies bind to antigens (association rate) and how long the bond lasts (dissociation rate). The affinity of produced antibodies for a specific antigen increases in germinal centres through a process called affinity maturation, during which B cells with higher affinities have a competitive advantage and get positively selected to differentiate to antibody-producing plasma cells. Our research shows that the mechanism by which B cells capture Ag affects GC dynamics and GC output with respect to B-cell receptor kinetics. Notably, in a mechanism where rupture of CC-FDC bonds is possible during Ag extraction, B-cell clones with low dissociation rates outcompete clones with high association rates over time. Understanding how B cells get selected in germinal centres could help to develop an optimised and effective immune response against a disease through vaccination for a fast-operating and long-lasting immune response.

## 1. Introduction

**Fig 1:**
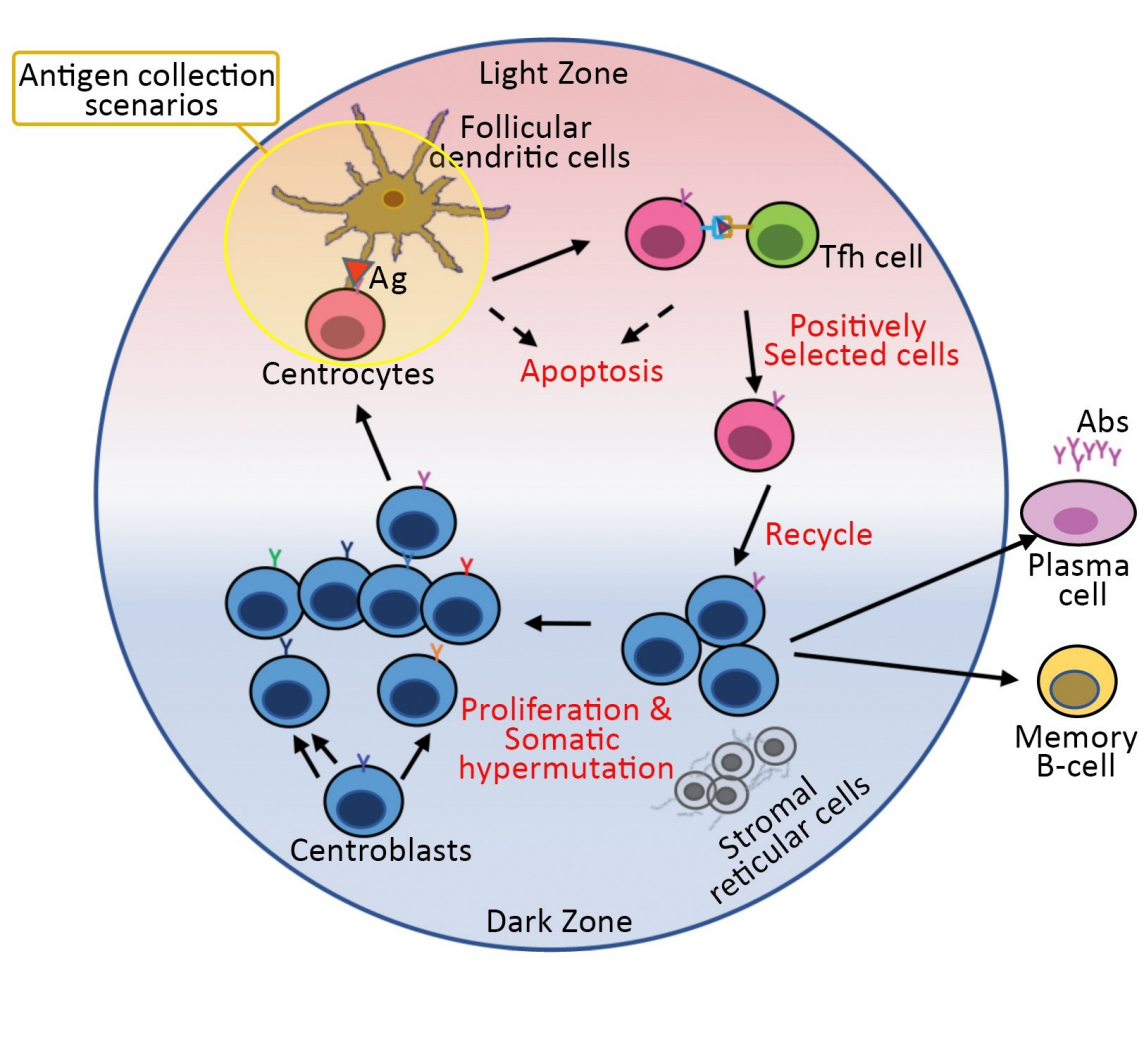
Schematic representation of a GC reaction. Reticular stromal cells express CXCL12 chemokine in the DZ (blue gradient), and FDCs express CXCL13 chemokine in the LZ (red gradient). CBs which are more sensitive to CXCL12 proliferate and change their affinity for the Ag through SHM in the DZ. Subsequently, CBs differentiate to CCs that move to the LZ because of their sensitivity to CXCL13 and collect Ag from FDCs. CCs internalise, process, and present the Ag peptides through MHCII molecules on their surface to interact with Tfh cells. CCs that cannot collect Ag and or cannot receive Tfh help due to competition die by apoptosis. Positively selected CCs recycle back to DZ for further proliferation and mutation or differentiate to MBCs or PCs.

Adaptive immunity is one of the vital defence mechanisms of the immune system in which high-affinity antibodies (Abs) are produced in response to specific antigens (Ag). The quality of interaction between an Ag epitope and an Ab paratope can be described by the affinity, which is a thermodynamic measurement used to rank the strength of reversible bimolecular interactions [1]. In the course of an adaptive immune response, the affinity of Abs increases through a process called affinity maturation that takes place in germinal centres (GCs). GCs are microanatomical structures developed within secondary lymphoid organs during the adaptive immune response [2] and consist of a light zone (LZ) and a dark zone (DZ) [3] (Fig 1). In the DZ, B cells which are called centroblasts (CBs), proliferate and gain new affinities for the Ag through somatic hypermutations (SHM) in their B-cell Receptor (BcR) genes [4]. BcRs are transmembrane proteins on the B-cell surface, comprised of CD79 and a membrane-bound Ab (immunoglobulin) that enables a B-cell to interact and bind with an Ag. Proliferation and SHM result in a pool of CBs with different affinities for the Ag. Subsequently, CBs differentiate to centrocytes (CCs) and move to the LZ to get positively selected in an affinity-dependent manner [5–7]. CCs collect and internalise Ag captured from a network of follicular dendritic cells (FDCs) [8,9], process, and present the resulting peptides in the form of peptide-MHC II complexes on their surface. Next, T follicular helper (Tfh) cells bind to the presented peptides, thereby providing survival signals to the B cells. Positive selection depends on the concentration of peptide-MHC II that B cells can present to Tfh cells [6] and, consequently, on the amount of Ag captured by the B cells. Low-affinity CCs that cannot collect (sufficient) Ag will receive no or only limited Tfh cell help and, consequently, are driven into apoptosis. Positively selected CCs eventually differentiate to memory B cells (MBC) or high-affinity long-lived plasma cells (PC) [10,11] or undergo further rounds of proliferation and mutation in the GC. Repeated proliferation, mutation, and selection lead to affinity maturation and production of high-affinity PCs that further secrete high-affinity Abs.

Affinity discrimination is the collective of mechanisms that provide high-affinity B cells with an advantage for collecting Ag and receiving support from Tfh cells. Affinity discrimination starts from the earliest stages of interaction between the BcR and Ag that lead to BcR signalling, spreading of the B-cell over the Ag presenting surface [12], formation of immune synapses [13,14], and initiation of endocytosis process and Ag collection [15,16]. BcR oligomerisation [17–20] and time-dependent BcR signalling in which BcRs gain signalling capability only after a certain time interval after Ag association [21] are proposed mechanisms for affinity discrimination. Oligomerisation and growth of BcR micro clusters are affinity-dependent events in which high-affinity BcRs have a higher chance of staying in bond with Ags and oligomerise [18].

The affinity of the BcR (membrane-bound Ab) for the Ag is inverse of the dissociation equilibrium constant K_D_ that is defined as [Ab][Ag]/[Ab-Ag], has the dimension of concentration, and is equal to the ratio of the dissociation rate (k_off_) and association rate (k_on_) constants (K_D_ = k_off_/k_on_) [22].

In an affinity-based selection model, B cells with higher affinities are favoured over B cells with lower affinities. However, since affinity is the ratio of the kinetic rates, equal affinity BcRs may correspond to different combinations of association and dissociation rates. Given CCs of equal affinity, two extreme cases of these combinations would be high association/dissociation rates (i.e., high on-off rate constants) or low association/dissociation rates (i.e., low on-off rate constants). The former leads to rapid binding of the CC with presented Ags but also rapid unbinding, while the latter results in forming tighter bonds at a price of slower association. Considering that the neutralisation capacity of produced Abs can be correlated to both on-rates and/or off-rates for different Ags, it is important to determine if such a selection mechanism could operate in the GC and how this would shape the composition of the memory B cells and plasma cells and finally affect the shape of adaptive immune receptor repertoire with respect to kinetics of produced Abs.

Although it is generally believed that selection in GC is based on affinity, this does not exclude that the selection mechanism operates on the level of k_on_ and or k_off_, resulting in the maturation of particular association and or dissociation potency of B cells along with affinity maturation [23–28]. Affinity maturation is believed to shift the Ag binding mechanism from an induced-fit to a lock-and-key binding model resulting in decreased k_on_ rates (since the Ab and Ag must be precisely positioned to bind) and decreased k_off_ rates (once bound, it will require more energy to rerelease the Ag) [29,30]. Experiments support this theory by showing that the entropy penalty of association [31] decreases over the course of maturation due to repeated immunisations. Moreover, molecular dynamics simulations show that Ab’s flexibility decreases during affinity maturation [32]. Repertoire analysis of anti-phOx Abs produced after primary immunisation and boosters at 6 weeks and 1 year showed an increase in affinity that corresponded with a decrease in dissociation rate constants [25]. However, observation of Abs with high dissociation rate constants and significantly high association rate constants in the repertoire led to the conclusion that selection in GC is not entirely based on tight binding of the BcR to Ag that is thermodynamically favourable, but also, rapid binding (high on-rate) of BcR to Ags which is kinetically favourable could lead to the selection of B cells. Other studies in humans [23,26] supported optimised dissociation rate constants during repeated immunisations but could not detect a significant change in association rate constants, while Sagawa and co-workers showed a decrease in association rate constants along with a decrease in dissociation rate constants with an overall improvement of affinity [27]. Moreover, some studies show that the efficiency of Ag presentation to T cells depends on the off-rates of BcRs [33,34].

Although none of these studies excludes the possibility that B-cell selection is driven by affinity, they do suggest an alternative scenario where selection is based on binding kinetics. However, mechanisms that would result in a bias towards clones with specific kinetic properties are unknown and not investigated. In the current work, using computational modelling, we show that such mechanisms may operate in the GC. Moreover, molecular dynamics simulations on the level of a single B-cell interaction with tethered Ags [20] have shown that both on- and off-rates affect affinity discrimination through the oligomerisation process by keeping off-rates constant and varying on-rates and vice versa. In another study [35], GC B cells were shown to make highly dynamic contacts with low-affinity Ags placed on planar lipid bilayers but were not able to form stable contacts, whereas, in interaction with high-affinity Ags, GC B cells had formed stable contacts through time in a punctuate pattern. In an affinity-based selection model, B-cell clones with high-affinity expand due to positive selection, recycling, and proliferation, while B-cell clones with lower affinities have less chance to survive. However, it has not yet been established whether there is a mechanism that facilitates clonal competition based on association and dissociation rates.

Computational modelling has been used for several decades to study the GC reaction. For example, these models have been used for studying clonal selection and maturation of the immune response [36,37], the kinetics of Ab-Ag binding [38,39], BcR-Ag interactions on the scale of a single cell [20,40–42], and the GC reaction with an affinity-based selection of B cells [5,43–45]. These models have resulted regularly in new hypotheses that can drive new experiments.

The simulations presented in this work aim to propose a putative mechanism for Ag collection that leads to a selection bias during the GC reaction for clones with specific kinetic properties. To simulate GC reaction, we extend an existing model that is used in many publications to develop GC reactions in silico [5]. The mechanism that we propose was guided by incomplete knowledge and theories about the kinetics of Ag collection and its synergy with the overall GC reaction.

In particular, we considered the interaction of the BcR with FDC-presented Ag on a clonal level. To do this, we modelled clonal competition between founder GC B cells with initially equal affinities but different association and dissociation rates and compared the evolution of these founder clones during a typical 21-day GC reaction.

Our simulations show a selective advantage for B-cell clones with low dissociation rates if we assume a mechanism in which the Ag collection process can be disrupted before being completed due to forces involved in the extraction process [14,46]. However, at the same time, these clones are not of the highest affinity. Moreover, a discrimination pattern was observed from the selection and differentiation of B cells to output cells in Scenario-1, which is suggestive of an existing discrimination mechanism between memory B cells and plasma cells based on kinetic rates of BcRs.

The proposed mechanism may inspire future experiments to investigate the role of BcR-Ag kinetics in GC selection in more detail.

## 2. Results

### 2.1 Overall setup of simulations

We performed a series of simulations for three scenarios (each repeated 30 times), starting with three founder B cells (clones) with low and identical affinities but different association/dissociation rates modelled as probabilities. Three clones were defined as Clone-L (low association/low dissociation), Clone-M (moderate association/ moderate dissociation), and Clone-H (high association/ high dissociation) to investigate the effect of low dissociation rate and or high association rate on GC selection and clonal evolution (Table 1). The effect of SHM is limited depending on clonality, so cells from Clone-L always have a fixed low-dissociation rate while Clone-H cells always have a fixed high-association rate. Hence, offspring of Clone-L could improve their affinities by increasing their association rates through SHM and getting positively selected, whereas offspring of Clone-H could improve their affinities by decreasing their dissociation rates through SHM. Initially, since B cells are of identical affinities, offspring of Clone-L will have a low association rate to presented Ags, whereas cells from Clone-H will have a high dissociation rate. Offspring from Clone-M represent cells between the two extreme cases. Eventually, cells from all clones could reach high association and low dissociation rates through affinity maturation.

**Table 1:** Initial affinity, association and dissociation rates of founder clones.

Clone	Initial rates and affinities			Effect of SHM
	Association	Dissociation	Affinity	
**Clone-L**	Low (P_a_ = 0.04)	Low (P_d_ = 0.0)	Low (P = 0.04)	Association
**Clone-M**	Moderate (P_a_ = 0.2)	Moderate (P_d_ = 0.8)	Low (P = 0.04)	Association/Dissociation
**Clone-H**	High (P_a_ = 1.0)	High (P_d_ = 0.96)	Low (P = 0.04)	Dissociation

P_a_, P_d_ and P represent probability of association, dissociation and affinity of CCs in the shape-space, respectively. The effect of SHM is different for each clone. For more detail, check the description of methods in section 4.4.

### 2.2 Affinity-based competition for Ag collection (Scenario-0, reference)

**Fig 2:**
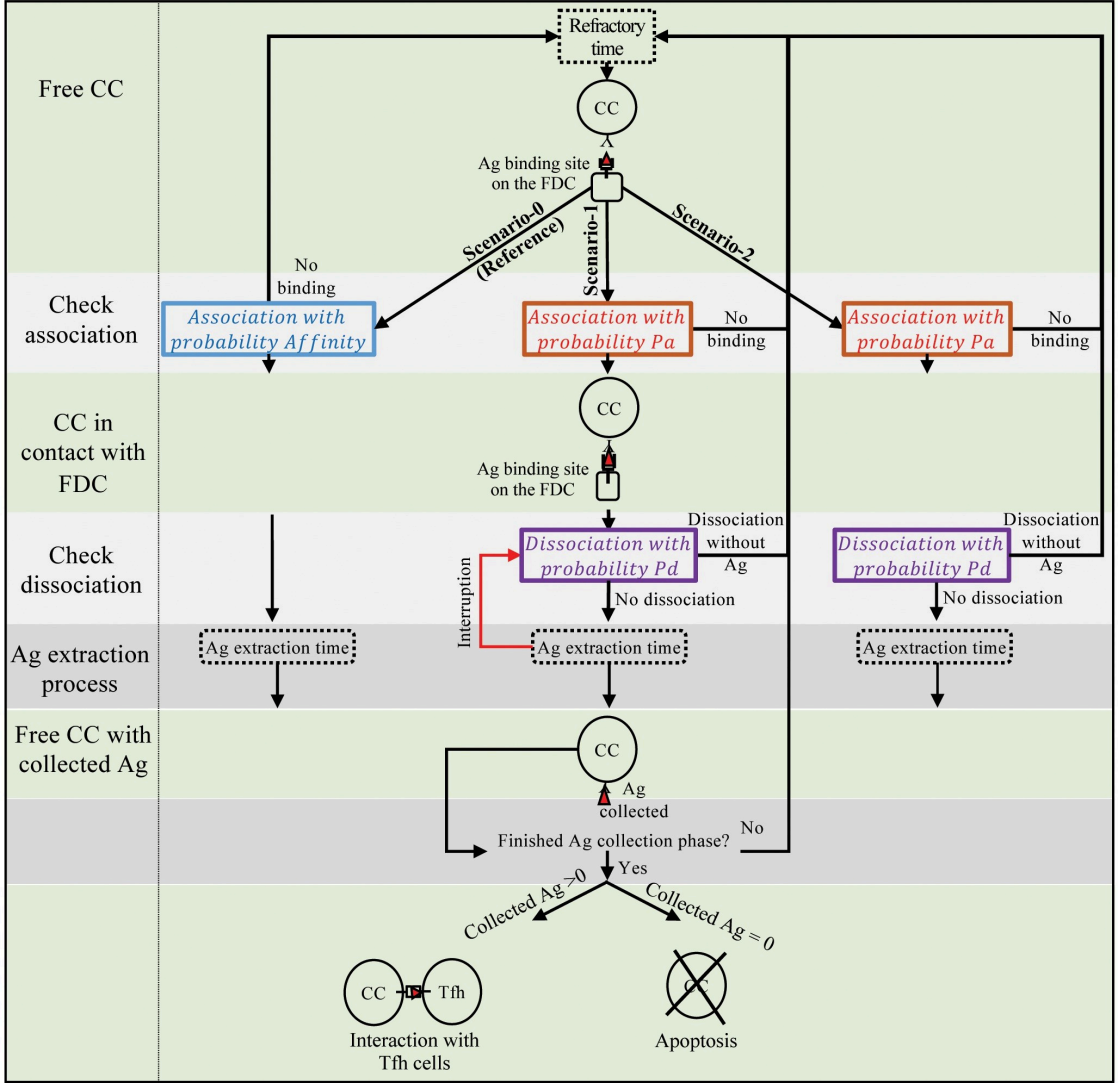
Schematic of Ag collection scenarios. Three mechanisms of Ag collection based on affinity and kinetic selection. The green rows denote the steps that the scenarios have in common. The starting point of each scenario is the event at which a free CC arrives at an Ag binding site. All scenarios end when the Ag collection phase is finished (last grey row), and the cell can engage in T-cell interactions. When no Ag is collected within 42 minutes, the CC will go into apoptosis. (A) Scenario-0: Competition for Ag depends on affinity directly. The CC’s probability of binding to Ag depends on the local Ag concentration (not shown in the figure) and CC’s affinity. After binding, the Ag extraction always starts directly. CC stays in bond until the Ag extraction process is finished after a period specified by a parameter ’Ag extraction time’ whereafter the CC captures the Ag. Subsequently, the CC may collect more Ag or engage in T-cell interactions. (B) Scenario-1: Competition for Ag collection depends on association (P_a_) and dissociation (P_d_) probabilities of CC that rely on the affinity. In this scenario, a CC associates to Ags presented on FDCs according to Ag concentration at the binding site and P_a_. In the next time step, the bond dissociates with a probability of dissociation (P_d_) or otherwise, CC initiates the Ag extraction process. During the Ag extraction process, the bond between CC and Ag still can get disrupted probabilistically (red arrow) at each time-step (dt = 0.002 h) with probability Pd that may lead to disruption of Ag extraction before it is fully complete, in which case the CC dissociates without obtaining Ag. Subsequently, if the bond between CC and Ag does not dissociate due to interruptions during the Ag extraction, CC collects the Ag and re-engages in another interaction. (C) Scenario-2: Similar to Scenario-1, only there are no interruptions after initiation of Ag extraction.

In the reference scenario (Fig 2, Scenario-0), the acquisition of Ag is solely dependent on the affinity, and GC B cells with higher BcR affinities will collect Ag more than B cells with low affinities [35]. Consequently, this gives higher affinity cells the advantage of receiving more help from Tfh cells than those with lower affinities [6] and getting positively selected. Therefore, Ag collection by B cells is defined as an event in which binding of the CC to Ag presented by the FDCs directly depends on the affinity and the Ag concentration at the binding site, resulting in an affinity-based B-cell competition for collecting Ag.

**Fig 3:**
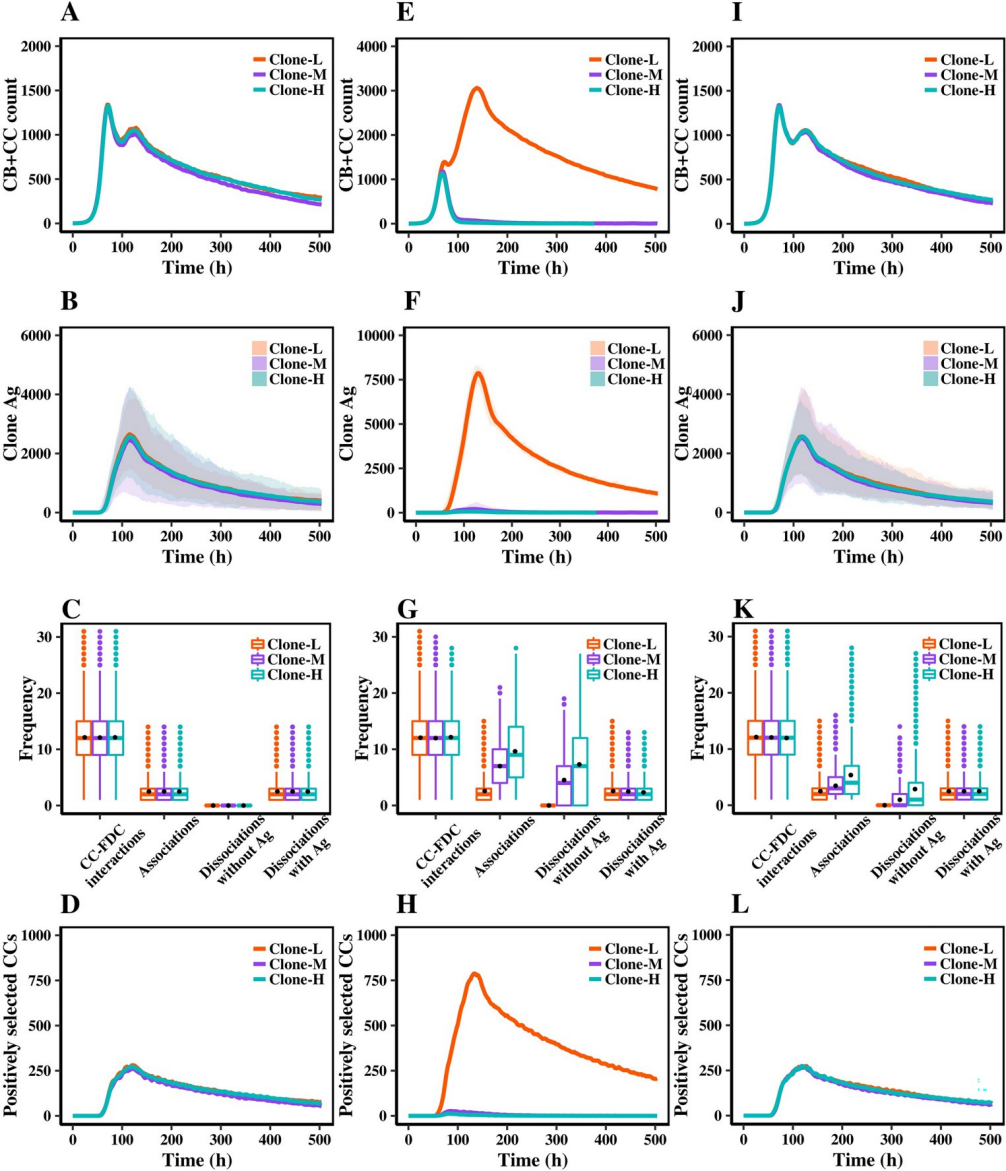
Dynamics of population and Ag collection phase. Results are obtained from 30 simulations. (A) The average of CB+CC counts for each clone in the reference scenario (Scenario-0). (B) Collected Ag for each clone during the GC reaction in the reference scenario. This consists of Ags collected by all CCs in each clone. Lines represent averages. The shaded area denotes the minimum and maximum values in all repeats. (C) Events during the Ag collection phase for CCs that attend the Tfh selection phase in the reference scenario. CC-FDC interactions denote the events at which the CC is located at an Ag binding site on a FDC. Boxplots are produced based on combined data of all simulations. Black dots represent average values per cell in each clone over all repeats. (D) The population of CCs that are positively selected by Tfh cells. Solid lines represent the average of all simulations. (E), (F), (G), and (H) represent the results of Scenario-1. (I), (J), (K) and (L) represent the results of Scenario-2. Panel descriptions are similar to that of the reference scenario.

We first aimed to confirm that affinity-based competition for Ag collection (Scenario-0; reference) resulted in the expected GC dynamics for a 21-day model of GC reaction. Since the initial affinities of the clones were identical to each other and the association/dissociation rates did not directly play a role in the Ag collection process, we did not observe any difference in overall population dynamics between the clones other than caused by the stochasticity of the model itself (Fig 3A). The first peak results from the clonal expansion phase of the GC reaction, and is reduced at the moment cells migrate to the light zone to go into apoptosis unless positively selected. The second peak is a result of a high positive selection rate that temporarily increases the number of cells. Population dynamics of the individual simulations are provided in S1 Fig.

Fig 3B shows that the amount of collected Ag by three clones was similar during the 21-day GC reaction. We investigated the Ag collection process for each clone in more detail by considering CCs that subsequently engage in Tfh cell interactions (Fig 3C). Consequently, CCs that did not collect Ag were not included in this analysis since these CCs became apoptotic and were removed from GC. The frequency of CC-FDC interactions (i.e., a CC occupies an Ag binding site on an FDC), the number of times a CC associated with Ag, the number of times a CC dissociated from FDCs without collecting Ag, and the number of times a CC dissociated from FDCs with collected Ag were similar between cells from the three clones.

Fig 3D shows the population of CCs that had been positively selected by Tfh cells. There was no difference observed between the population size of positively selected CCs from three clones.

We conclude that in the reference scenario (Scenario-0), where competition for Ag collection depended directly on affinity and association/dissociation probabilities did not play a role, the three clones with equal initial affinities showed similar dynamics during the Ag collection phase, collected similar amounts of Ag, and consequently received equal Tfh cell help that led to similar population dynamics and equal evolution of three clones.

### 2.3 Interruptions during Ag extraction cause a selective advantage for B-cell clones with lower dissociation rates (Scenario-1)

Here we propose a mechanism operating in the GC and contributing to B-cell affinity discrimination. We make the binding of Ag to the BcR explicitly dependent on the association rate while also allowing unbinding, prior to initiation of Ag extraction, according to the dissociation rate (Fig 2, Scenario-1). This mechanism assumes that association does not immediately result in Ag extraction, but rather to initiate the process of Ag extraction, BcRs must have reasonably low dissociation rates that permit BcR oligomerisation after initial association and subsequently lead to signalling and collection of Ag. Consequently, clones with a too high dissociation rate will not be able to initiate Ag extraction.

Moreover, recent studies show that B cells use mechanical forces for extracting tethered Ags through exerting pulling forces on BcRs by myosin II contractility [16,35,46–51]. Repeated exertion of pulling forces on BcRs by B cells results in rupture of weaker (higher off-rates) bonds between the Ag and BcR that do not endure the stress and have been suggested to be responsible for more stringent affinity discrimination by negatively regulating Ag collection [14,46]. Thus, these forces potentially disrupt the extraction process of bond Ags before it is completed. Therefore, inspired by the mechanism of force application, we implemented interruptions during the Ag extraction process. We are not modelling the exact mechanism of force application in scenario-1 but instead represent the rupture of bonds probabilistically and assume the rupture can occur at any moment during the Ag extraction process after it is initiated and before completion. As a result, and in contrast to scenario-0, CCs that are associated with Ag and have initiated extraction process could dissociate from FDCs during the Ag extraction process without capturing the Ag.

Scenario-1 simulations showed a clear difference between the population dynamics and Ag collection profile of three clones compared to the reference scenario. Clone-L was dominant in cell counts (Fig 3E) and amounts of collected Ag (Fig 3F). Clone-M and Clone-H were not dominant after day 6 of the GC reaction in any of the 30 individual simulations (S2 Fig). The population dynamics in Scenario-1 (Fig 3E) look different with respect to the reference scenario (Fig 3A). In Scenario-1, the implementation of the new mechanism forms a stronger selection criterion in GC. As a result, the initial population growth is smaller than the reference scenario that lowers the initial peak. The composition of the B-cell population has changed from three clones forming population in similar sizes in the reference scenario to Clone-L forming the majority of population in Scenario-1. Clone-L also collected more Ag in Scenario-1 in comparison to the reference scenario, while Clone-M and Clone-H could not collect Ag due to competition with Clone-L B cells. The existence of this competitive effect was further verified by simulating Scenario-1 for only Clone-M, where in the absence of Clone-L B cells, cells from Clone-M take up more Ag and Clone-M expands (S7 Fig).

As before, the frequency of CC-FDC interactions was similar between clones since it is mainly dependent on CC movement. However, the frequency of association events and dissociations clearly differed between CCs from the three clones (Fig 3G). Even though Clone-H and Clone-M had a higher association frequency because of their higher association rates, they also exhibited more dissociations without collecting Ag due to their higher dissociation rate. In contrast, Clone-L engaged in fewer bindings due to lower association rates but had a lower rate for dissociating and, hence, more chance of collecting Ag. Clone-H showed a slightly lower average per cell compared to Clone-M, and Clone-M compared to Clone-L (Fig 3G, black dots). The values are mentioned in S1 Table. Even though the average collected Ag per cell was not significantly different between cells from three clones, the total collected Ag by Clone-L was significantly higher than the other two clones (Fig 3F). Note that apoptotic CCs which could not collect Ag were not included in this analysis. This implies the number of cells that could collect Ag from Clone-L was significantly higher than that of the other two clones, and most of the CCs from Clone-H and Clone-M could not collect any Ag at all and therefore could not receive help from Tfh cells and went to apoptosis. Therefore, the majority of positively selected CCs were from Clone-L (Fig 3H).

We conclude that a mechanism in which the Ag extraction process can be disrupted due to rupture of the bond between CCs and FDCs gives a selection bias towards clones that strongly bind (low off-rate) to the Ag. At the same time, clones with fast binding (high on-rate) but less strong binding (high off-rate) could not grow in GC. A high association rate did not contribute much to overcome interruptions during the Ag extraction. Instead, a low dissociation rate was a necessity for collecting Ag. Therefore, more cells from Clone-L were positively selected compared to the other two clones, resulting in further reproliferating and dominance of cells from this clone.

To confirm that the selective advantage of Clone-L in Scenario-1 originated from the interruptions during the Ag extraction process, we performed an additional set of simulations in which the bond between the BcR and Ag could not get interrupted once the extraction process is initiated (Fig 2, Scenario-2). Without these interruptions, the three clones showed similar population dynamics (Fig 3I) and Ag collection profiles (Fig 3J). The population dynamics of repeated simulations are provided as a supplementary figure (S3 Fig). The frequency of CC-FDC interactions did not show a significant change as it is mainly dependent on cell motility. However, the association frequency of Clone-M and Clone-H decreased (Fig 3K) compared to Scenario-1. This is because in Scenario-1, interruptions during Ag extraction could cause disruption of bond before collecting the Ag, and therefore, CCs had to re-engage in Ag collection more frequently for capturing the Ag. By removing interruptions, CCs had a higher chance of collecting Ag in each interaction, and therefore, the frequency of dissociations without collected Ag decreased in Scenario-2 compared to Scenario-1. Consequently, the decrease in dissociation rates led to a decrease in the frequency of associations since fewer reengagements were needed for capturing Ag compared to Scenario-1. Moreover, the average frequency of dissociations with Ag was equal between three clones (Fig 3K, black dots). The population of positively selected CCs from three clones were equal (Fig 3L), as also observed in the reference scenario. Hence, we conclude that the selective advantage towards Clone-L that existed in Scenario-1 was originated from the interruptions during Ag extraction since without such interruptions, we observed three clones evolved similarly in GC.

### 2.4 Interruptions during Ag extraction affects the distribution of produced plasma and memory B cells population and increases levels of affinity maturation in GC

**Fig 4:**
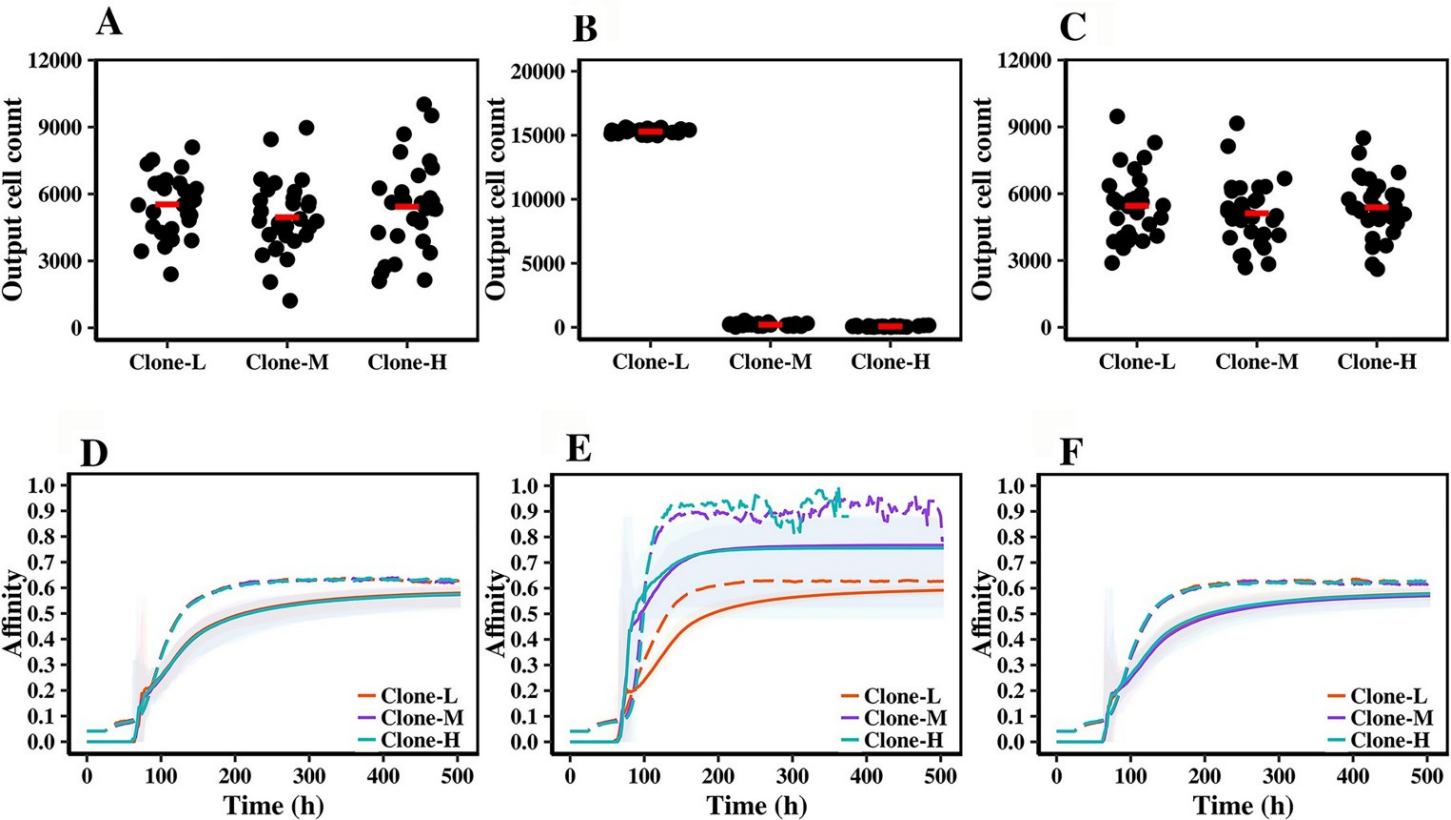
OCs production and affinity maturation. Results are obtained from 30 simulations. (A), (B) and (C) represent produced OCs from each clone in the reference scenario, Scenario-1 and Scenario-2, respectively. Each dot represents OCs produced in a single simulation. Red lines denote the average. (D), (E) and (F) represent the average affinity of living GC B cells (dashed lines) and the cumulative average of produced OCs (solid lines) belonging to each clone in the reference scenario, Scenario-1 and Scenario-2, respectively. Shaded areas indicate the maximum and minimum of OC’s average affinity in 30 simulations.

To further investigate the effect of introduced probabilistic ruptures during Ag extraction on GC output, we looked at the population of produced output cells (OCs), consisting of both plasma cells and memory B cells, and affinity maturation of three clones in GC. Fig 4A shows the population of OCs produced from each clone in each of the 30 repeats in the reference scenario. A similar number of OCs was produced from three clones during the 21-day GC simulation in the reference scenario. However, in Scenario-1, most of the produced output cells (OC) were from Clone-L (Fig 4B). Clone-L had lower dissociation rates compared to Clone-M and Clone-H, and subsequently, a higher chance of Ag collection, which led to an increase in Tfh cell help and, therefore, virtually all produced OCs were derived from Clone-L. The origin of this bias towards Clone-L was the interruptions during Ag extraction, as removing these interruptions in Scenario-2 resulted in an equal number of OCs being produced from three clones (Fig 4C).

In the reference scenario (Fig 4D), three clones reached equal levels of affinity due to affinity maturation. Interestingly, in Scenario-1, both GC B cells and OCs belonging to Clone-M and Clone-H showed a higher average affinity (Fig 4E) in comparison to the reference, whereas Clone-L showed a similar level of affinity maturation as the reference scenario. The cause of these increased affinity levels for Clone-M and Clone-H was the interruptions during the Ag extraction process, as removing them in Scenario-2 restored affinity maturation levels (Fig 4F) to the reference. Note that OCs in our model do not die and, therefore, the average affinity of the OCs population (Fig 4) includes affinity of all OCs generated from t = 0 up to the current time point. However, the average affinity of GC B cells is calculated for existing GC B cells at each time-point and therefore average affinity of OC population and GC B cells cannot be compared with each other in this figure.

In the reference scenario and Scenario-2, since initiation of Ag extraction led to capture of Ag without any interruptions, the competition between clones for capturing the Ag was only limited to initiating the extraction process. However, introducing probabilistic ruptures due to interruptions in Scenario-1 formed an extra step of competition for Ag collection between clones in which initiating Ag extraction was not enough for capturing Ag, and a low dissociation rate was necessary to endure probabilistic ruptures during extraction. The increased affinity maturation for Clone-M and Clone-H resulted from the more stringent competition for Ag collection in Scenario-1, i.e., cells from these clones could only survive if their affinity was sufficiently high, and thus their dissociation rates were sufficiently low. Thus, these two clones were under strong selective pressure for survival. However, Clone-L did not show any increment in affinity maturation levels in comparison to the other two clones or in comparison with the other two scenarios because this clone had a fixed low dissociation rate (P_d_ = 0) and, therefore, cells from Clone-L never dissociated from Ag during the extraction process due to interruptions (Fig 3G). Therefore, there was no extra selection pressure on Clone-L in comparison to other scenarios to further improve affinity. However, since the competition for associating with Ag and initiating the extraction process still existed between cells from this clone, Clone-L’s association rate evolved over time, which led to an increase in the affinity of this clone. The average affinity of GC B cells in each simulation of the reference scenario, Scenario-1 and Scenario-2 are provided in supplementary figures (S4, S5 and S6 Figs, respectively).

We conclude that as probabilistic rupture of CC-Ag bonds due to interruptions during Ag extraction improved affinity discrimination by increasing dissociation rates and making the Ag capturing more difficult for clones that did not have initially optimal dissociation rates, the affinity maturation levels increased, an effect that was suggested to result from force application by B cells for extracting Ag [14,46].

## 3. Discussion

It is generally believed that positive B-cell selection in the GC is based on affinity, which is strongly supported by the observation that the affinity of B cells improves during the GC reaction. However, affinity is a thermodynamic quantity that results from association and dissociation kinetics. Therefore, it cannot be excluded that the GC selection mechanism operates on the level of these individual rates. Indeed, several (vaccination) studies have reported a bias in the production of antibodies during an immune response towards specific association or dissociation rates [23,25,26]. This suggests that optimisation of affinity might be accompanied by optimisation of kinetics to, for example, achieve maximum pathogen neutralisation capacity. Understanding if and how Ab binding kinetics plays a role during affinity maturation might prove to be helpful for the design of vaccines. Several studies investigated the correlation of association and dissociation rate constants with neutralising potency. In one study, neutralising capacity was mainly shown to correlate with association rate constants [52]. Moreover, it was shown that discrimination between heterologous and homologous peptides of a model epitope was dependent on association rate constants suggesting that on-rates being an important subject of maturation during an immune response [28]. However, in a more recent study, it was suggested that neutralising capacity correlates with dissociation rate constants [53]. Although none of these studies focused on a single-GC, we assume that optimising kinetic rates along with affinity finds its origin in processes facilitated by single or multiple subsequent GC reactions over a longer period of time. These studies suggest that selection pressure may not (only) operate at the level of affinity but also at the level of the underlying association and/or dissociation rates. However, as far as we are aware of, molecular and cellular mechanisms underlying selection bias for kinetic properties have never been investigated experimentally in the context of a GC reaction. Consequently, precise mechanisms, if existent, remain to be established. Our research proposed a mechanism that potentially drives kinetic selection, and therefore might provide a starting point for further experimental research.

Our simulations showed that an affinity-based mechanism (Scenoria-0) or modelling binding and unbinding according to association and dissociation rate (Scenario-2) does not discriminate between clones with different kinetic properties. Consequently, in these scenarios, cells with fast association rates and cells with slow dissociation rates co-exist in the GC and achieve similar affinity and output cells. However, Scenario-1 demonstrated that a mechanism in which binding and unbinding are modelled with the corresponding kinetic constants and, in addition, Ag capturing can be interrupted before completion, leads to a selective advantage for cells with low dissociation rates, i.e., cells that strongly bind Ag. Consequently, the PC and MBC compartments will also be enriched for those cells. Interestingly, these strong binding cells do have lower affinities compared to co-existing cells with higher association and dissociation rates. However, since they have a lower dissociation rate, they possess a better selection chance with respect to the Ag collection mechanism used in Scenario-1 (Fig 4E). In a more recent study on SARS-CoV-2 antibodies, it was observed that an antibody with decreased affinity compared to its germline affinity but improved (decreased) dissociation rates could in fact, be subject to positive selection in the GC [53].

Another interesting observation in Scenario-1 was the composition of output cells with respect to their kinetic properties, suggestive of a discrimination pattern between memory B cells and plasma cells. It has been shown that memory B cells are generally of lower affinity [54,55] but are also less specific to allow recognition of future variants of the Ag compared to plasma cells which are of higher affinities and more specific. In the current model, we do not distinguish between memory B cells and plasma cells but the results suggest that cells with higher on-rates and high off-rates mainly differentiate to OCs in the first couple of days (memory B-cells) while at later stages, they are outcompeted by plasma cells that are of very low dissociation rates. One could hypothesise cells that can bind easily to Ags (high on-rates) but cannot extract much (high off-rates), receive high BcR signals but low CD40 signals, while cells that have low off-rate values and could extract more Ag compared to the aforementioned cells also receive higher CD40 signals and differentiate to plasma cells.

Our model and simulations have several limitations. Firstly, the kinetics and affinity maturation and GC dynamics are likely to be dependent on the specific Ag that triggers the immune response. However, with the current GC model, we cannot simulate specific Ags. Secondly, given the results of previous (vaccination) studies that led to different conclusions with respect to the selection bias for clones with specific kinetic properties [23,25–28], different or additional mechanisms may be operating during the GC reaction that affect the GC output with respect to kinetic properties of BcRs such as Ab feedback [56,57]. Perhaps, depending on external cues delivered by the Ag or otherwise, the GC can decide for or switch between different selection mechanisms to optimally steer an immune response. Although our disruption mechanism is based on known biology, we are currently investigating other mechanisms. For example, in the current model, we kept Θ, the ratio between the association and dissociation distances fixed. Consequently, the effect of SHM for each clone was constant and never changed. Therefore, we are currently developing a model in which SHM directly affects Θ, increasing the heterogeneity of the clones in the simulation. Other mechanisms might include B-cell velocity [58], chemokine sensitivity, or Ag concentration, which would all affect the association rate. Thirdly, the three clones defined in our model represent limited but also extreme choices for the association and dissociation rates for the three defined clones. This does not reflect the true heterogeneity of clones in a GC and, therefore, future simulations should take such heterogeneity into account, e.g., using a mechanism as proposed above. Fourthly, the high probability of bond disruption in Scenario-1 is not based on experimental data and, therefore, could also have been assigned a lower value, which could restore the balance between kinetic selection based on the rapid binding and thermodynamic selection based on tight binding. Scenario-2 is an extreme example of this, with the interruption probability set to zero. Finally, our results are difficult to compare to the aforementioned (vaccination) studies because we modelled a single GC reaction while these experimental studies measured the Ab response after, for example, repeated vaccinations over longer periods of time and are likely the composite result of multiple GC reactions. Moreover, these studies measure affinity and kinetic rates of a limited number of free Abs in solution, which may differ from the affinity (avidity) of the membrane-bound BcR for the FDC presented Ag. Hence, the presented results remain qualitative, and it is not possible to quantitatively compare these with experimental data.

It is worth pointing out the difference between kinetic rates of interaction between free Ab-Ag in solution and membrane-bound Ab-Ag interaction should be considered in experimental studies since the former follows 2D kinetics while the latter is described with 3D kinetics. We could not find an experimental study for BcRs; however, in case the of TcR-pMHC binding, it has been shown that 2D on-rates are faster compared to 3D on-rates while 3D dissociation rates are higher than 2D [59,60]. Moreover, a unified mathematical framework has been proposed for addressing 2D/3D kinetic differences in the case of TcR-pMHC [61] that has been adopted to investigate BcR interaction with FDC-presented Ag concerning 2D/3D kinetic rates [62].

In conclusion, we propose a mechanism operating in the GC and inspired by affinity discrimination which is the collective of mechanisms that provide high-affinity B cells with an advantage for Ag collection and Tfh cell support. We assumed that Ag binding by the B-cell is solely based on the association kinetics while unbinding is determined by dissociation. Crucially, we demonstrate that allowing disruption of Ag collection before completion results in a selective advantage for clones with low dissociation rates.

Experimental follow-up is required to validate and complement our findings and to acquire a much better understanding of the molecular, cellular and physical processes involved in the Ag collection and to establish the role of kinetics in B-cell selection. To falsify and or validate the current results, one can measure the Ag uptake of two GC B-cell populations with equal affinities but different association/dissociation rates from FDC presented Ags [63]. The observed difference in the amount of collected Ag, if any, could clarify to what extent our results can represent real biology. Moreover, one could reproduce the competition of clone-M -L and -H in an animal model. Selected antibodies specific for a given antigen and reproducing characteristics of the 3 clones should be used to generate transgenic paired-BCRs knock-in mice. These B cells can be further adoptively transferred together in a WT/or B cell deficient recipient mouse, following immunisation, compartment distribution (spleen, blood, bone marrow), phenotype, and maturation of the three clones should be studied [64].

## 4. Methods

### 4.1 GC agent-based model

We extended a well-established agent-based (ABM) spatiotemporal representation of GC [5,65] to model Ag collection based on reaction rates of CCs interactions with presented Ags in GC. Here, we briefly describe the relevant components of the model. The GC is modelled in a three-dimensional spherical simulation space with a radius of 160 micrometres with a volume of ~17 nanolitres and discretised by a lattice constant of 5.0 micrometres. Pre-calculated steady-state CXCL12 and CXCL13 chemokine concentration gradients produced by reticular stromal cells and follicular dendritic cells (FDCs) are imposed on the grid. Agents consist of GC B cells with centroblasts (CB) or centrocytes (CC) phenotype, T follicular helper (Tfh) cells, FDCs, and output cells (OC) without distinguishing between memory B cells (MBC) and plasma cells (PC). Each simulation models a GC reaction over a 21day period (typical GC lifetime) with a time step of 7.2s. Cell motility is implemented as a directed random walk with different cell types moving with different velocities and directions according to the chemokine gradients. After several rounds of proliferation and accumulating SHMs, CBs differentiate to CCs and move toward FDCs due to their sensitivity for CXCL13, collect Ag, and subsequently interact with Tfh cells to become positively selected. Tfh cells help the neighbouring CCs with the highest Ag concentration by providing survival signals during an interaction. Positively selected CCs recycle to the DZ to further proliferate, differentiate to OCs, or engage in the next GC cycle (Fig 1). Differentiation of CCs to OCs is based on the asymmetrical and symmetrical distribution of Ag upon cell division in which cells that end up with a large portion of Ag after division differentiate to OCs while cells with the lower portion of Ag or symmetrically distributed Ag remain in GC and continue further rounds of selection. This mechanism is implemented according to the original model and resulted in closer agreement with experimental data of transzonal migration rates [5]. All parameters used in these simulations are from the original model unless otherwise stated.

### 4.2 Affinity and somatic hypermutation

Affinity is defined in terms of an equilibrium reaction in which BcR and Ag form a complex:

$$BcR+Ag\overset{k_{on}}{\underset{k_{off}}{⇌}}BCR-Ag$$

with the equilibrium dissociation constant (K_D_) defined in terms of concentrations and inversely related to affinity:

$$\frac{1}{Affinity}=K_{D}=\frac{\left[BcR][Ag\right]}{\left[BcR-Ag\right]}=\frac{koff}{kon}$$ (1)

Here k_on_ has the unit of [M^-1^ s^-1^] and depends on the concentration, k_off_ has the unit of [s^-1^], and consequently, affinity has the unit of concentration [M^-1^].

Kinetic rates can be modelled as a probability by considering the exponential distribution, which is a probability distribution of time between events in a Poisson process:

$$f(t)=k_{on}e^{-k_{on}t}$$ (2)

The probability for binding to occur within a certain time period (τ) then can be estimated with the cumulative probability distribution function:

$$P(X\leq \tau )=\int_{0}^{\tau }f(t)dt=1-e^{-k_{on}\tau }$$ (3)

Similar probabilistic representations have, for example, been used to model single interactions of membrane-bound and or free immunoglobulins with Ags [20,41,42,66,67].

SHMs are point mutations, changing the BcR genes and leading to variations in the structure and affinity of BcRs, therefore, changing the association and dissociation rate constants. However, it is not an easy task to calculate the affinity/kinetics of the interaction without the structural information of BcR and Ag complexes and their free states [1]. Particularly in the case of GC simulations, the number of mutations and the lengthy time of GC reaction (21 days) makes affinity prediction/calculation based on structural information computationally expensive. To reduce the computational burden, we model these interactions on a cellular scale using the shape-space concept, which is based on the assumption that an Ab evolves towards a protein structure that is complementary to the Ag structure resulting in strong binding, i.e., high affinity [36,68]. In the context of the ABM, each CC and the Ag are represented in an abstract shape-space grid, and affinity is defined as the L1-norm (d) between a CC and the Ag located in the shape space. The distance (*d*) is translated to an affinity value between 0 and 1 using:

$$Affinity=e^{-\frac{d^{2}}{\Gamma^{2}}}$$ (4)

Here Γ is the affinity weight function’s width (Γ = 2.8) based on experimental data [68]. These affinity values represent shape-scores for each CC concerning the specific Ag and are defined between 0 and 1 that can be interpreted as binding probabilities.

The Ag has a fixed position in the shape-space. SHM results in a change in the position of the CC in the shape-space by one grid point, which changes the distance (*d*) between the CC and Ag and, consequently, the CC affinity for the Ag either increases or decreases depending on the decrease and or increase in the distance respectively. The one-step jump in the shape-space results in a set of discrete affinities.

### 4.3 Reference (Scenario-0): Affinity-based competition for Ag collection

In the reference scenario, Ag collection is modelled as an affinity dependent process identical to the original model (Fig 2, Scenario-0). During the Ag collection phase, CCs have a fixed time-window during which they can interact multiple times with FDCs to collect Ag. CC-FDC interactions are defined as a one-step event in which free CCs, when they arrive at an Ag binding site, bind to Ag with a probability based on the local Ag concentration and affinity. Association of CCs to FDCs initiates the Ag extraction process. The CCs stay in bond for the duration of Ag extraction, which is defined probabilistically (P_Finishing Ag extraction_ = 0.04 per dt; dt = 7.2 s), and return to their free state after capturing the Ag. The CC always captures Ag after association. There is a short refractory time (72 seconds) after each interaction during which CCs cannot interact with FDCs. This is to prevent CCs from repeatedly trying Ag collection at one binding site [65,69]. Dissociation is not explicitly implemented other than the stochastic length of binding. Therefore, the probability of acquiring Ag through a single CC-FDC interaction (P_Ag_) is equal to the probability that CC binds to Ag (P_Association_) and in this scenario depends on the affinity of CC in the shape-space and the concentration of Ag in the binding site:

$$P_{Ag}=P_{Association}=Affinity*P_{C}$$ (5)

P_C_ has a value between 0 and 1 depending on the concentration of Ag at the binding site (C_Ag_) and the saturation level of Ag for a single CC (S_Ag_ = 20 unit of Ag). Therefore, when there is no Ag at the binding site P_C_ = 0 and when the Ag concentration is higher than the saturation level of CC, P_C_ = 1; otherwise, P_C_ = C_Ag_/S_Ag_.

This implementation results in a competition for Ag that directly depends on the affinity in which CCs with higher affinities, have a higher probability of collecting Ag in each interaction and therefore gather greater amounts of Ag in time.

### 4.4 Scenario-1: kinetic-based competition for Ag collection with probabilistic rupture of bonds during Ag extraction

In this scenario, Ag collection is modelled by explicitly considering the association and dissociation rates underlying affinity and modelling these rates as probabilities. To model association and dissociation probabilities, we extend the concept from the previous scenario by introducing probabilities P_a_ and P_d_:

$$Affinity=P_{a}*(1-P_{d})$$ (6)

Here, P_a_ and P_d_ represent the association and dissociation probabilities of each CC according to the affinity in the shape-space that can be changed by SHM. With this definition, an increase in affinity due to SHM could be translated to an increase in association probability or a decrease in dissociation probability. Similarly, a decrease in affinity could translate to an increase in dissociation probability or a decrease in association probability. Therefore, possible effects that SHM could have on off-on rates could be implemented by this approach.

Then from Eqs (4) and (6) we have:

$$Affinity=e^{-\frac{d^{2}}{\Gamma^{2}}}=P_{a}.(1-P_{d})=e^{-\frac{x^{2}}{\Gamma^{2}}}.e^{-\frac{y^{2}}{\Gamma^{2}}}$$ (7)

**Fig 5:**
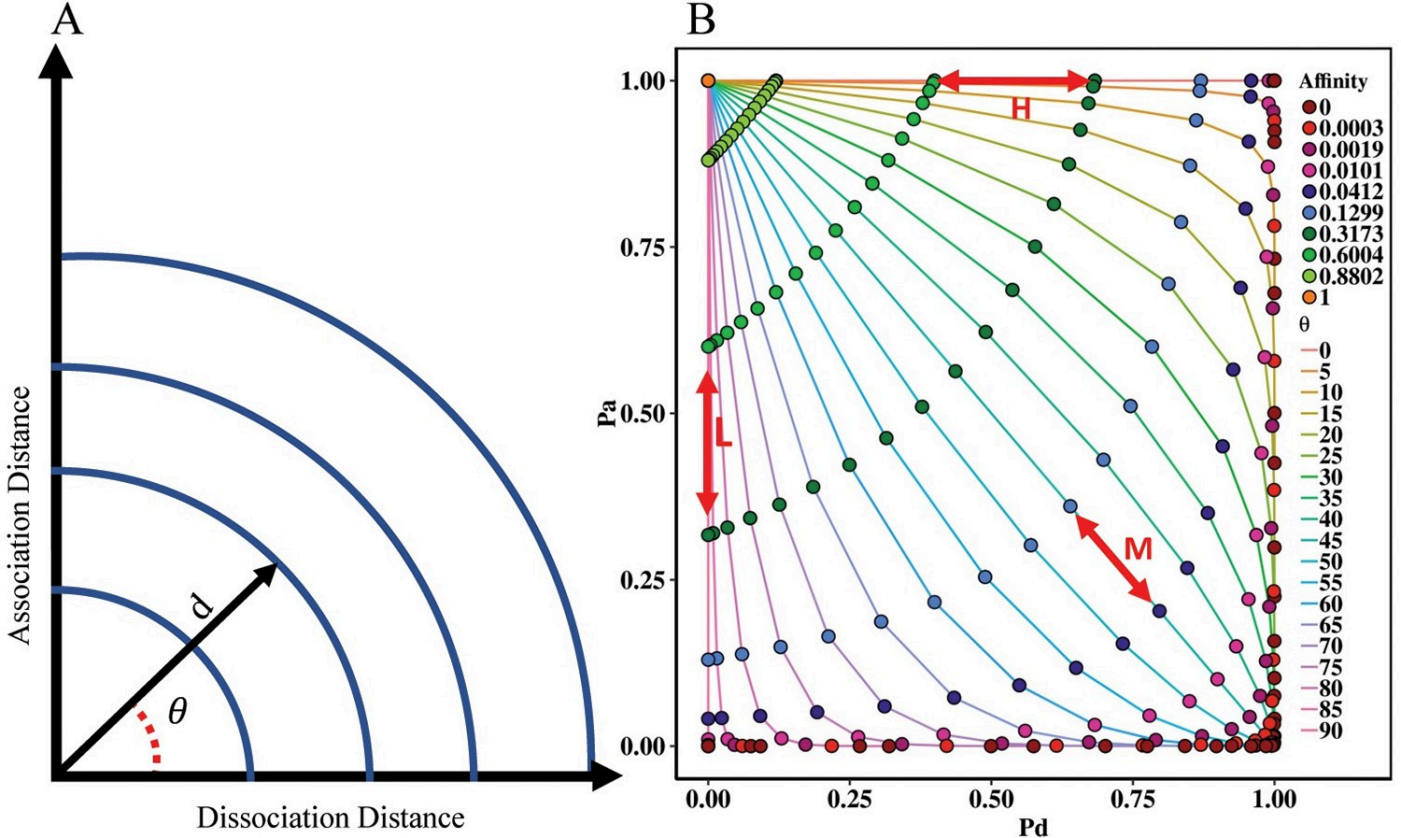
Correlation between P_a_, P_d_, affinity and Θ plotted for discrete affinity and Θ values. (A) Schematic of correlation between distance *d* in the shape-space and the corresponding association and dissociation distances defined by Θ. The blue iso-affinity curves denote combinations of distances that result in identical affinities. (B) The correlation between P_a_-P_d_ and affinity of CC in the shape-space. Red arrows represent the three clones defined by different Θ values. SHM moves cells from each clone along the lines that represent specific Θ values resulting in lower or higher affinities (represented by the points). Affinities below 0.0003 are considered as 0.

In which the distance d is now decomposed in x and y representing the association and dissociation distances respectively, which by definition are also on a scale from 0 to 1 and, therefore, can be interpreted as probabilities (Fig 5A). This decomposition is facilitated by introducing a parameter Θ that reflects the ratio at which we decompose distance d into the association and dissociation distances. Now we derive:

$$P_{a}=e^{-\frac{d^{2}\sin^{2}\theta }{\Gamma^{2}}}$$ (8)

$$P_{d}=1-e^{-\frac{d^{2}\cos^{2}\theta }{\Gamma^{2}}}$$ (9)

Fig 5A shows the correlation between the distance in the shape-space (d), association distance, dissociation distance and Θ. Fig 5B shows the numerical correlation between P_a_, P_d_, affinity and Θ.

The change in the P_a_ and P_d_ depends on the change in the distance (*d*) and Θ. Since Θ is fixed, the P_a_ for Clone-H (Θ = 0) is not affected by SHM. Similarly, since Θ = 90 for Clone-L, the P_d_ for this clone is unaffected by SHM (Fig 5B, red arrows; Table 1). Clone-M, with Θ = 45, can change both probabilities upon SHM.

The probability of associating to Ag presented by FDCs (P_Association_) that depends on both the concentration of Ag and the association probability of a CC according to shape-space then is:

$$P_{Association}=P_{a}.P_{C}$$ (10)

Each CC, when at the binding site, binds to an Ag according to association probability (P_Association_). In the next time-step, the CC can dissociate according to dissociation probability (P_Dissociation_) and return to the free state without collecting Ag, or it will stay in contact with the FDC and initiate Ag extraction process. We assume that the Ag extraction process can be disrupted after initiation, and consequently, CC moves to the free state and try to re-engage in another interaction. This is implemented by introducing interruptions during the extraction of Ag at each time-step (dt = 7.2 s) that could cause disruption of Ag extraction and dissociating without Ag. If Ag extraction is completed successfully, CC moves to free state with collected Ag and could initiate another interaction.

Then the probability of acquiring Ag through each CC-FDC interaction (P_Ag_) would be:

$$P_{Ag}=P_{Association}*(1-P_{Dissociation})*P_{SI}$$ (11)

Where P_*Dissociation*_ depends on the dissociation probability of CC according to shape-space (P_*Dissociation*_ = P_d_), and P_SI_ is the probability of surviving interruptions without the bond getting ruptured due to interruptions that is inversely correlated to P_d_. For simplicity, we assume P_SI_ = (1-P_d_)^N^, where N, is the number of interruptions, and since we introduce an interruption at each time-step, it depends on the time of Ag extraction.

### 4.5 Scenario-2: Ag extraction process without interruptions

In scenario-2 (Fig 2, Scenario-2), we removed the interruptions during the Ag extraction process. Therefore, the probability of collecting Ag presented by FDCs through each interaction becomes:

$$P_{Ag}=P_{Association}*(1-P_{Dissociation})$$ (12)

### 4.6 Deviation from the original model

The ABM of LEDA [5] was reproduced according to [65] and all the parameters used are borrowed from the original model except parameter Θ that is introduced in this paper. However, in all three scenarios, the dynamic mutation probability (DMP) was not included in ABM. The DMP implies that the mutation probability of B cells decreases as their affinity increases. Without DMP, the average affinity of living GC B cells drops to ~60%, while with DMP it restores to more than 90%.

### Software

ABM of GC is written in C++. All analyses were done in R. Software and parameter settings are available from GitHub: https://github.com/EDS-Bioinformatics-Laboratory/GC_from_Affinity_to_Kinetics.

## Supporting information

S1 Fig Population dynamics of clones for Scenario-0 in 30 simulations. The population of CBs+CCs for each clone in each of the 30 simulations in the reference scenario.
(TIF)

S2 Fig Population dynamics of clones for Scenario-1 in 30 simulations. The population of CBs+CCs for each clone in each of 30 simulations in Scenario-1.
(TIF)

S3 Fig Population dynamics of clones for Scenario-2 in 30 simulations. The population of CBs+CCs for each clone in each of 30 simulations in Scenario-2.
(TIF)

S4 Fig Affinity maturation of clones for Scenario-0 in 30 simulations. The average affinity of existing B cells and cumulative average of produced OCs in the reference scenario.
(TIF)

S5 Fig Affinity maturation of clones for Scenario-1 in 30 simulations. The average affinity of existing B cells and cumulative average of produced OCs in Scenario-1.
(TIF)

S6 Fig Affinity maturation of clones for Scenario-2 in 30 simulations. The average affinity of existing B cells and cumulative average of produced OCs in Scenario-2.
(TIF)

S7 Fig Results for simulations of Scenario-1 with only Clone-M. (A) The average population dynamics of CB+CC over 30 simulations. (B) The average number of positively selected CCs in 30 simulations. (C) The average value of collected Ag by Clone-M in 30 simulations. The shaded area shows the minimum and maximum collected Ag by this clone over time in 30 simulations. (D) Box plots of frequency of interactions for all CCs attending the Tfh-cell selection phase in 30 simulations. (E) The average affinity of existing cells from Clone-M in GC (dashed-line) and the cumulative average of produced OCs (solid-line) over 30 simulations. (F) The number of produced OCs in 30 simulations.
(TIF)

S1 Table Average values of events during Ag collection phase provided in Fig 3C, 3G and 3K. The average value of each event during the phase of Ag collection, for three clones in three scenarios.
(DOCX)

S1 File Data tables of three scenarios. Simulation data of three scenarios used to produce Figs 3 and 4, and S1–S7 Figs.
(ZIP)

S2 File Code used to produce figures. R scripts used for production of Figs 3–5, and S1–S7 Figs.
(ZIP)
