# Supplementary figures and images for: From affinity selection to kinetic selection in Germinal Centre modelling
Source: PLoS Comput Biol. 2022 Jun 3;18(6):e1010168. doi: 10.1371/journal.pcbi.1010168 (PMC9200358; doi:10.1371/journal.pcbi.1010168)

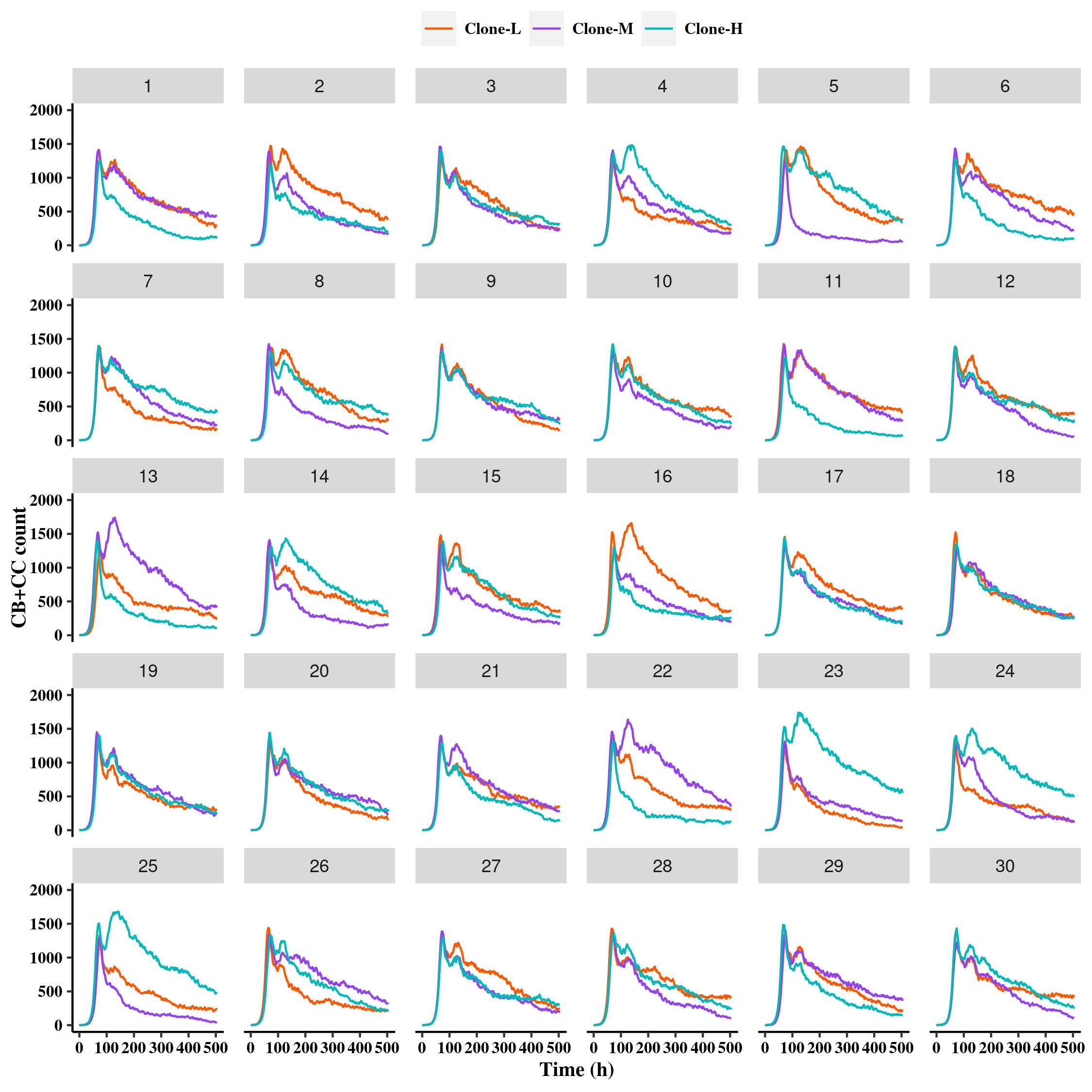

Supplement: S1 Fig — The population of CBs+CCs for each clone in each of the 30 simulations in the reference scenario. (TIF) [file pcbi.1010168.s001.tif]

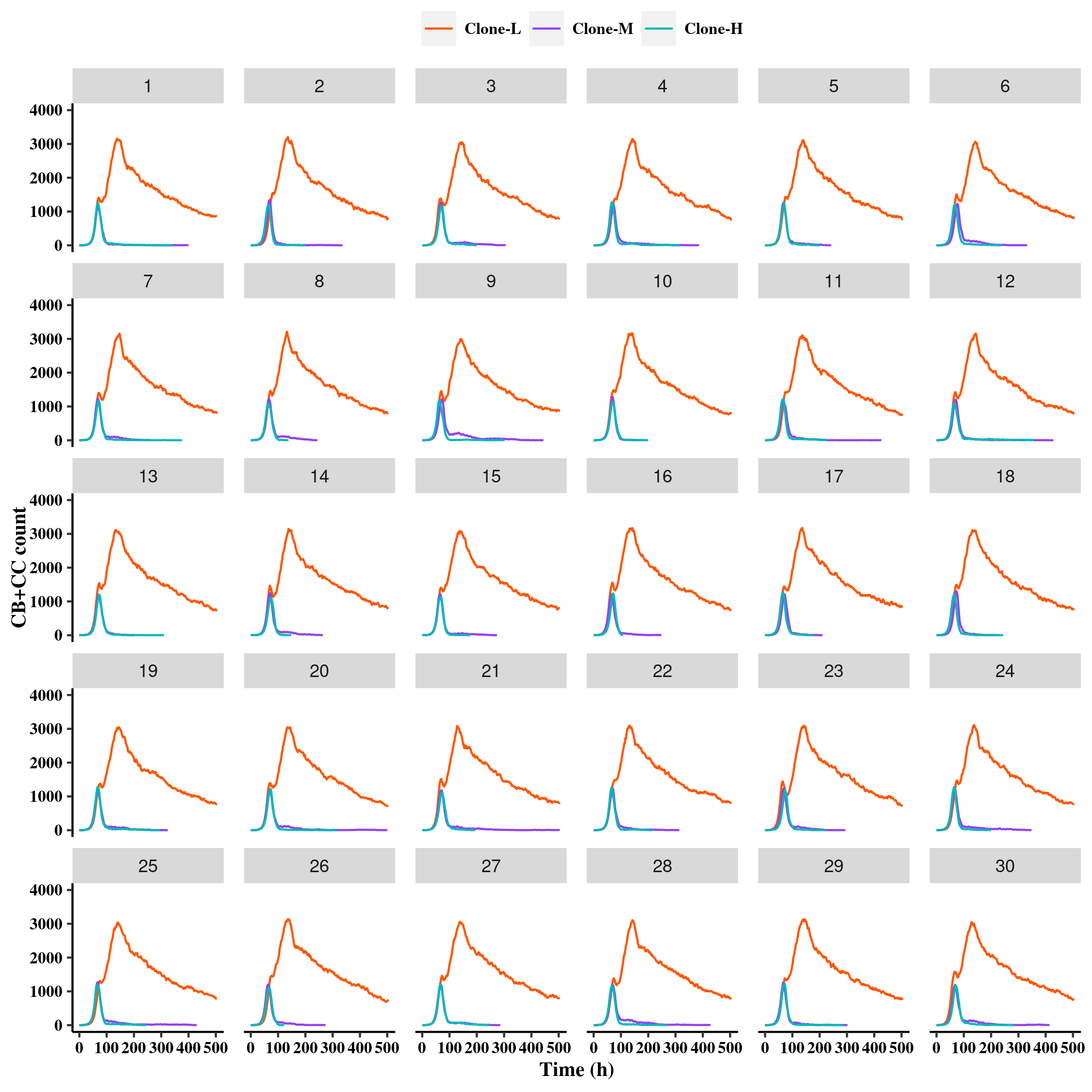

Supplement: S2 Fig — The population of CBs+CCs for each clone in each of 30 simulations in Scenario-1. (TIF) [file pcbi.1010168.s002.tif]

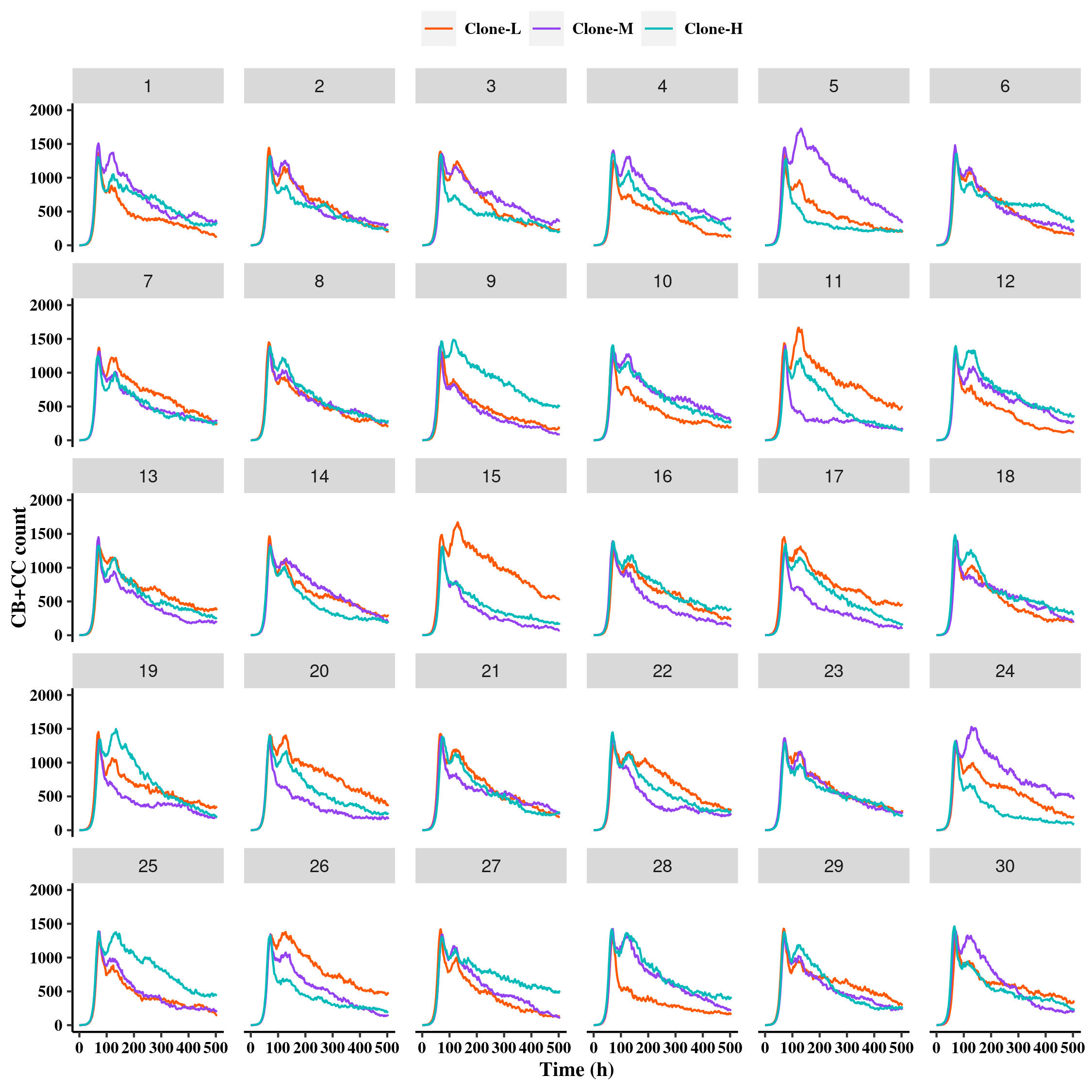

Supplement: S3 Fig — The population of CBs+CCs for each clone in each of 30 simulations in Scenario-2. (TIF) [file pcbi.1010168.s003.tif]

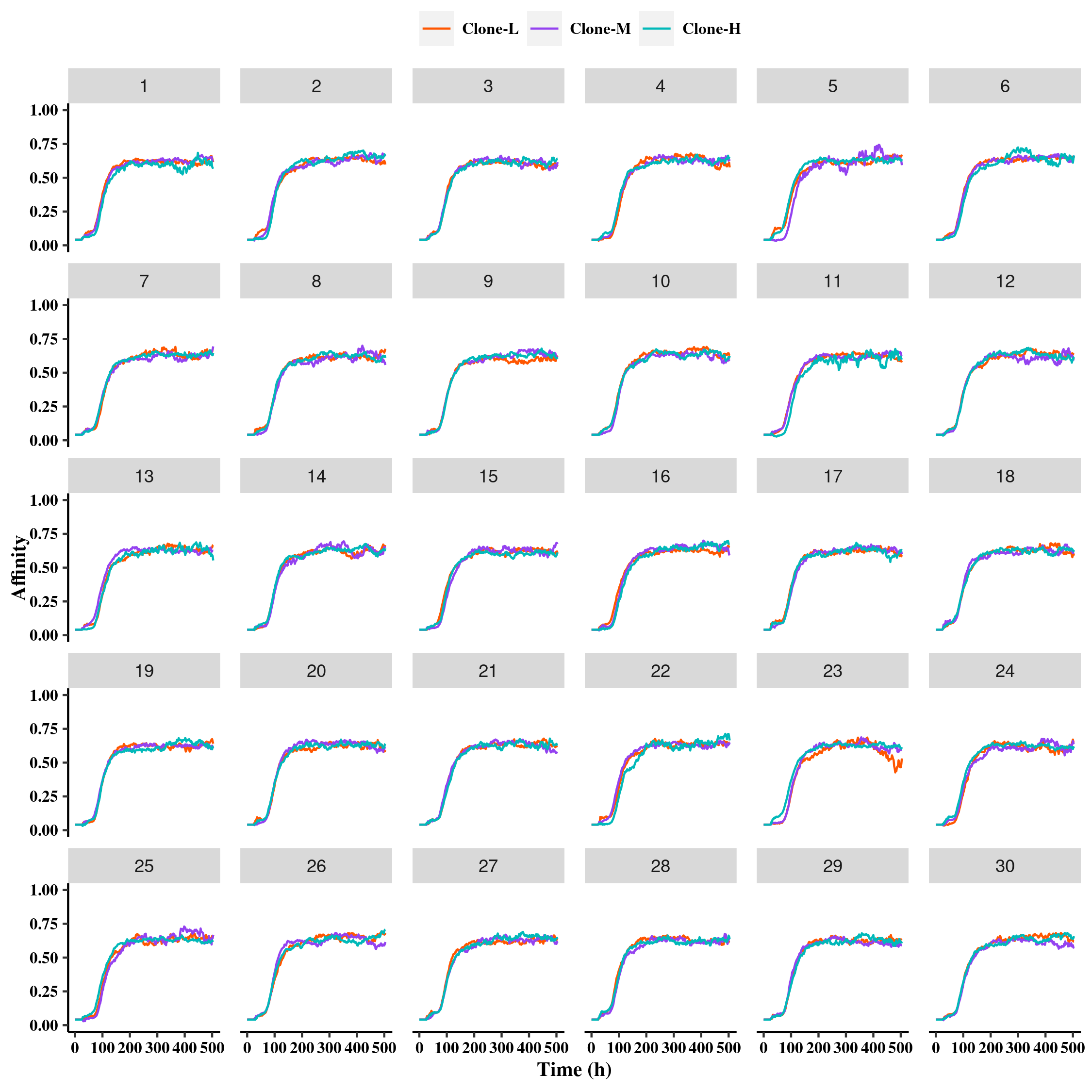

Supplement: S4 Fig — The average affinity of existing B cells and cumulative average of produced OCs in the reference scenario. (TIF) [file pcbi.1010168.s004.tif]

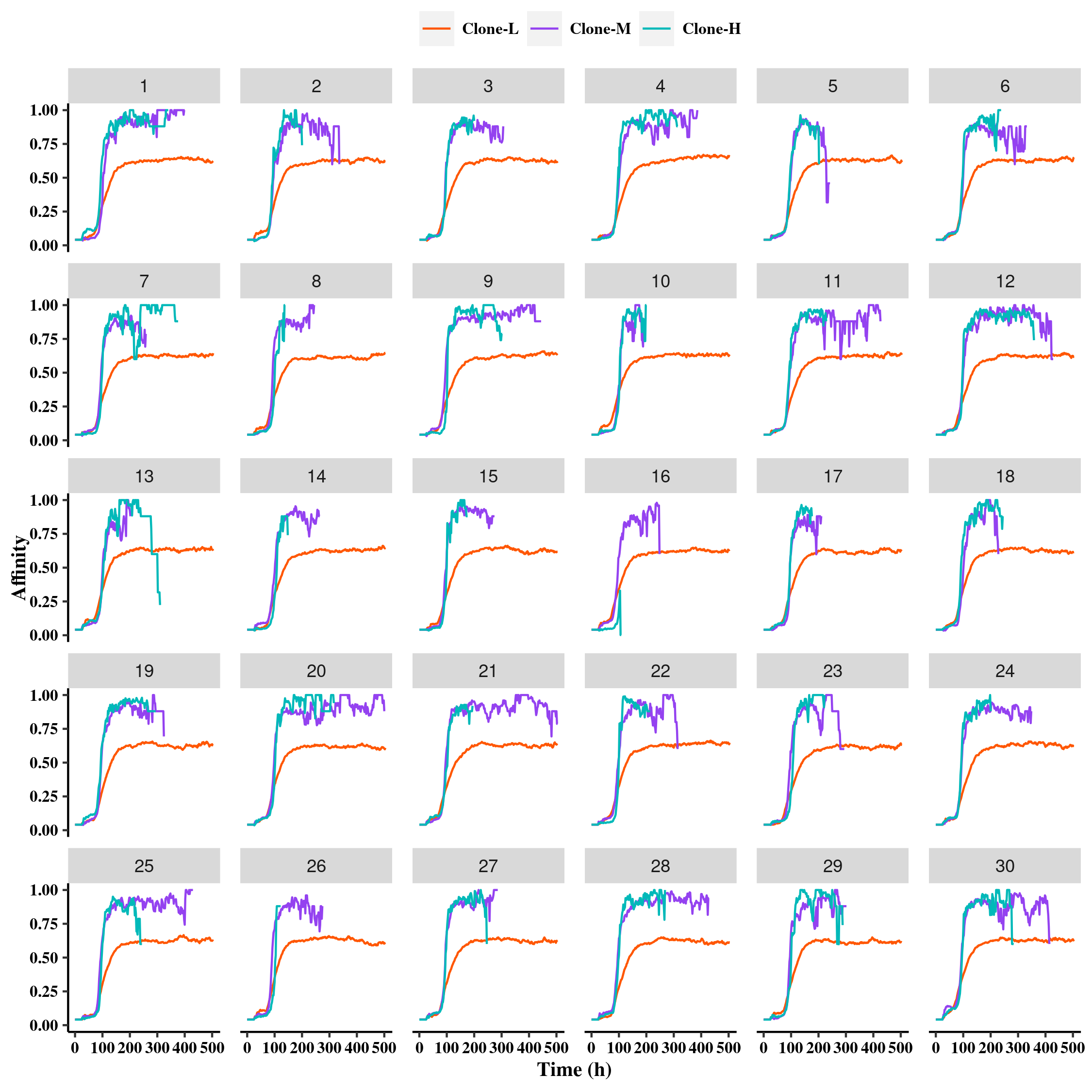

Supplement: S5 Fig — The average affinity of existing B cells and cumulative average of produced OCs in Scenario-1. (TIF) [file pcbi.1010168.s005.tif]

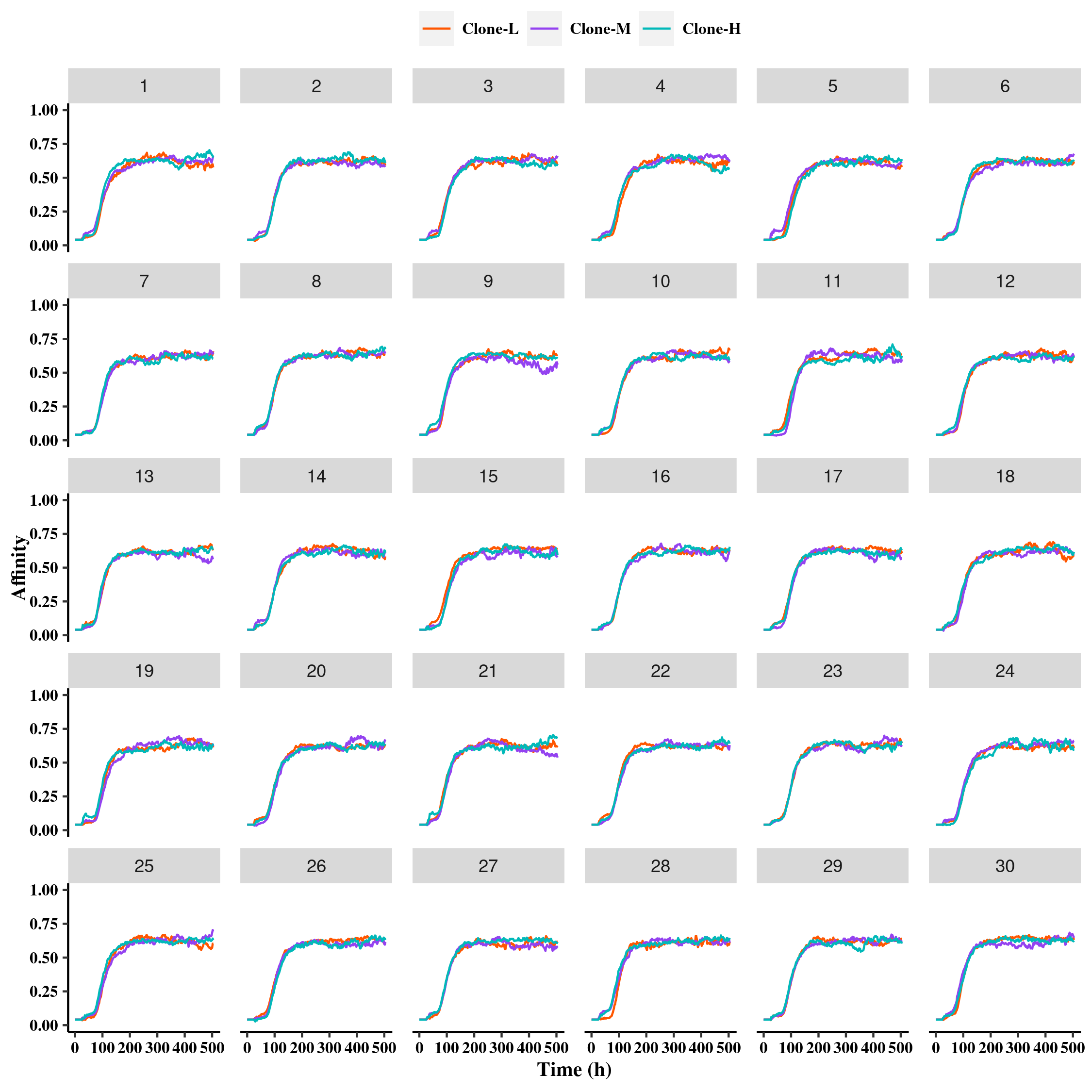

Supplement: S6 Fig — The average affinity of existing B cells and cumulative average of produced OCs in Scenario-2. (TIF) [file pcbi.1010168.s006.tif]

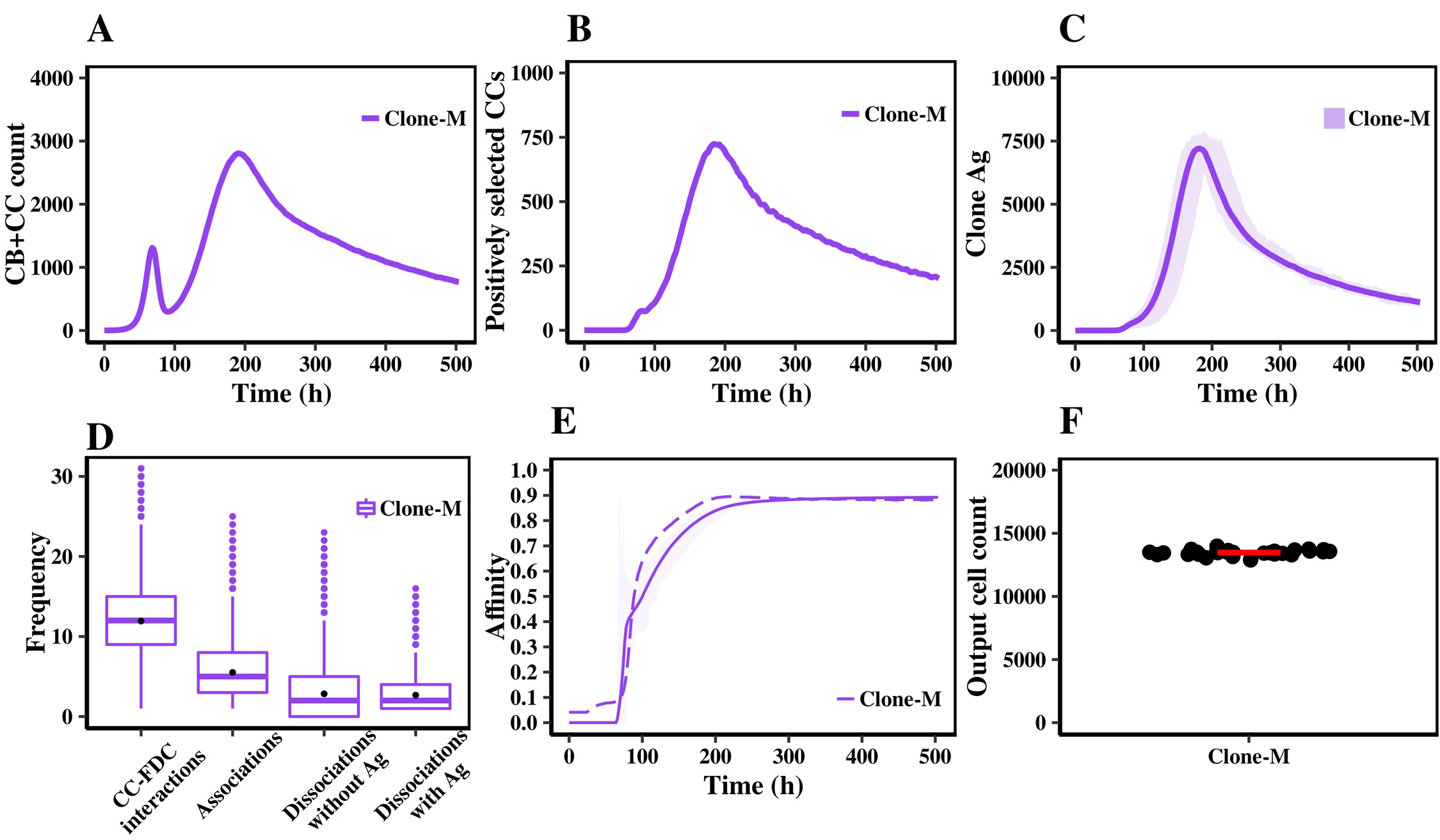

Supplement: S7 Fig — (A) The average population dynamics of CB+CC over 30 simulations. (B) The average number of positively selected CCs in 30 simulations. (C) The average value of collected Ag by Clone-M in 30 simulations. The shaded area shows the minimum and maximum collected Ag by this clone over time in 30 simulations. (D) Box plots of frequency of interactions for all CCs attending the Tfh-cell selection phase in 30 simulations. (E) The average affinity of existing cells from Clone-M in GC (dashed-line) and the cumulative average of produced OCs (solid-line) over 30 simulations. (F) The number of produced OCs in 30 simulations. (TIF) [file pcbi.1010168.s007.tif]
