# Supplementary material for: From affinity selection to kinetic selection in Germinal Centre modelling
Source: PLoS Comput Biol. 2022 Jun 3;18(6):e1010168. doi: 10.1371/journal.pcbi.1010168 (PMC9200358; doi:10.1371/journal.pcbi.1010168)
Supplement: S1 Table — The average value of each event during the phase of Ag collection, for three clones in three scenarios. (DOCX) [file pcbi.1010168.s008.docx]

**S1 Table**. **Average values related to Fig 3C, 3G and 3K.**

| Clone | Scenario | Frequency of CC-FDC interactions | Frequency of association | Frequency of dissociation without Ag | Frequency of dissociation with Ag |
| --- | --- | --- | --- | --- | --- |
| Clone-L | Reference | 12.123747 | 2.498696 | 0 | 2.498696 |
| Clone-M | Reference | 12.093261 | 2.499010 | 0 | 2.499010 |
| Clone-H | Reference | 12.126038 | 2.488153 | 0 | 2.488153 |
| Clone-L | SC-1 | 12.1 | 2.57 | 0 | 2.57 |
| Clone-M | SC-1 | 12.0 | 7.00 | 4.52 | 2.48 |
| Clone-H | SC-1 | 12.1 | 9.63 | 7.30 | 2.33 |
| Clone-L | SC-2 | 12.1221003 | 2.4947725 | 0 | 2.4947725 |
| Clone-M | SC-2 | 12.0581920 | 3.4556773 | 0.9723711 | 2.4833062 |
| Clone-H | SC-2 | 11.9571083 | 5.3654368 | 2.8709932 | 2.4944436 |
